# Assessment of the tau protein concentration in patients with tick-borne encephalitis

**DOI:** 10.1007/s10096-018-03447-1

**Published:** 2019-02-05

**Authors:** Piotr Czupryna, Barbara Mroczko, Sławomir Pancewicz, Paweł Muszynski, Sambor Grygorczuk, Justyna Dunaj, Karol Borawski, Magdalena Róg-Makal, Renata Świerzbińska, Joanna Zajkowska, Maciej Kondrusik, Anna Moniuszko-Malinowska

**Affiliations:** 1 Department of Infectious Diseases and Neuroinfections, Medical University in Białystok, Żurawia 14, 15-540 Białystok, Poland; 2 Department of Neurodegeneration Diagnostics, Medical University in Białystok, Jerzego Waszyngtona 15A, 15-269 Białystok, Poland; 3 Department of Invasive Cardiology, Medical University in Białystok, M. Skłodowskiej-Curie 24A, 15-276 Białystok, Poland

**Keywords:** Tick-borne encephalitis, Tau, Neurodegeneration

## Abstract

There have been suggestions that tick-borne encephalitis (TBE) may cause neurodenenerative changes in the brain. The aim of this study was the assessment of the tau protein concentration in cerebrospinal fluid (CSF) of patients with different clinical forms of TBE. The concentration of tau protein in CSF was determined using Fujirebio tests (Ghent, Belgium) in 35 patients with TBE: group I—patients with meningitis (*n* = 16); group II—patients with meningoencephalitis (*n* = 19). None of the patients reported any neurodegenerative disorder that could affect the results of the study. The control group (CG) consisted of 10 patients in whom inflammatory process in central nervous system was excluded. Tau protein concentration in CSF before treatment did not differ significantly between the examined groups, while its concentration was significantly higher in encephalitis group than in CG after 14 days of treatment. Significant increase in tau protein concentration after treatment was observed in both examined groups. The comparison between the group of patients who fully recovered and patients who presented with persistent symptoms on discharge showed significant differences in tau protein concentration before and after treatment. ROC curve analysis indicates that CSF tau protein concentration before treatment may predict complicated course of the disease with 90.9% specificity and 80% sensitivity, while after treatment, specificity became 72.7% and 71.4% for sensitivity. Correlation analysis showed that in TBE patients (both meningoencephalitis and meningitis groups), CSF pleocytosis before treatment correlated negatively with tau protein concentration in CSF. (1) Neurodegeneration process is present in TBE encephalitis. (2) Tau protein concentration may be used as a predictor of complicated course of TBE.

## Introduction

Tick-borne encephalitis (TBE) is an infectious CNS disease caused by tick-borne encephalitis virus (TBEV) of Flavivirus genus, transmitted by Ixodes ticks. There are between 200 and 300 cases registered per year in Poland. The majority of cases is noted in the Podlaskie Province.

TBE may take different clinical courses from asymptomatic to life-threatening and/or causing permanent neurological and cognitive deficits. We can differentiate ***three*** distinct clinical forms of TBE: meningitis, meningoencephalitis, meningoencephalomyelitis [1, 2].

In our previous studies***,*** we observed that neurodegenerative process is present in the course of TBE, as there was an increased production of neuron***-***specific enolase (NSE) in CSF [3].

The next step of our research is the assessment of a potential role of ***tau—***another marker of neurodegeneration.

Tau release may be caused by neuroinflammation. It is a microtubule-associated protein, predominantly expressed in the neurons, closely associated with the proper functioning of the cytoskeletal network in terms of microtubule assembly. Tau protein in the cerebrospinal fluid (CSF) is considered as an important biomarker of several disorders of the central nervous system (CNS) where axonal damage is contemplated, e***.***g.***,*** MS***;*** other inflammatory neurological diseases include ADEM (acute disseminated encephalomyelitis) [4–6].

There are reports of increased tau concentrations in CSF of patients with viral or bacterial neuroinfections, and it has been suggested that it could be the marker for parenchymal involvement [7, 8].

Detailed aims:

1. Evaluation of tau concentration in patients with TBE in cerebrospinal fluid before and after treatment
2. Comparison of tau concentration in patients with CNS inflammation and patients without CNS involvement
3. Comparison of tau concentration in patients with meningitis and meningoencephalitis
4. Comparison of tau concentration in patients with and without sequelae

## Material and methods

Tau concentration was measured in CSF of 35 patients treated in the Department of Infectious Diseases and Neuroinfections of the Medical University of Bialystok between the years 2013 and 2016 because of TBE.

Patients were divided into two groups depending on clinical course of the disease:

Group I—patients with meningitis (*n* = 16: 5 women, 11 men aged between 27 and 76 years; mean—54.2 ± 12.53 years old). Patients diagnosed with meningitis did not present any neurological findings. Group II—patients with meningoencephalitis (*n* = 19: 8 women and 11 men aged between 29 and 79 years; mean—48.12 ± 12.18 years old). Meningoencephalitis was diagnosed on the basis of consciousness disturbances and/or focal neurological findings.

There were no patients with meningoencephalomyelitis hospitalized during the time of our research. None of the patients had been vaccinated against TBE. Disease was diagnosed based on the clinical picture, presence of inflammatory parameters in the CSF, and specific antibodies present in serum and CSF. TBE antibody titer was measured with Enzygnost Anti-TBE/FSME Virus (IgG, IgM) Siemens test. A case of TBE was diagnosed according to EAN rules.

None of the patients reported any neurodegenerative disorder that could affect the results of the study. Patients were treated with 0.25 g/kg 15% Mannitol per dose 2–4 times a day and, if necessary, with NSAIDs (ketoprofenum). The treatment lasted for 4–7 days and had no effect on the study as sample 1 was taken before drug administration and sample 2 over 1 week after drug withdrawal.

The control group (CG) consisted of 10 patients (4 women, 6 men aged between 27 and 76 years; mean—51.5 ± 12.34 years old). These patients were admitted to the hospital because of headache and the CSF examination excluded inflammatory process. Additionally, they had no history of neurodegenerative disorders. Brain MRI images of these patients did not reveal any significant abnormalities.

Tau concentration in CSF was determined using Fujirebio tests (Ghent, Belgium).

Tau concentration was measured in CSF on admission (sample 1) and 14 days after admission (sample 2). Tau concentration ratio between sample 2 and sample 1 (tau 2/tau 1 ratio) was calculated.

One month after discharge, all the patients had a follow-up examination in the outpatients’ department for potential sequelae presence.

Sequelae were observed in 14 patients (6 woman, 9 men aged between 29 and 79 years). Two of these patients belonged to group I, 12 patients to group II. These patients presented with persistent headaches, tremors, ataxia, paresis, hearing impairment, and psychiatric disorders.

Patients voluntarily agreed to participate in the study and gave their written informed consent. The study was approved by the Local Bioethics Committee of the Medical University of Bialystok (number R-I-002/9/2018).

The statistical analysis was performed using STATISTICA 10. Groups were compared with Kruskal-Wallis, Mann-Whitney *U* test, Wilcoxon-matched pair test, and receiver operating characteristic curve (ROC) tests. Correlations were measured by the Spearman rank test.

## Results

The groups did not differ significantly as far as age and sex are concerned.

In group I (meningitis), in sample 1, the mean pleocytosis was 102 ± 66.34 cells, mean protein concentration—65.98 ± 21.12 mg/dl. In sample 2 (after 14 days), the mean pleocytosis was 55.36 ± 18.6 cells, mean protein concentration—65.14 ± 30.76 mg/dl.

In group II (meningoencephalitis), in sample 1, the mean pleocytosis was 135.6 ± 96.5 cells, mean protein concentration—73.74 ± 32.01 mg/dl. In sample 2 examination (after 14 days), the mean pleocytosis was 49.72 ± 32.98 cells, mean protein concentration—80.52 ± 47.08 mg/dl.

No significant differences were observed in pleocytosis or protein concentration between the groups.

Tau concentration in sample 1 did not differ significantly between the examined groups, while tau concentration in sample 2 was significantly higher in meningoencephalitis group than in CG (Table 1). There were no significant differences in tau 2/tau 1 ratio between groups I and II.

**Table 1:** Comparison of tau concentration in three groups (tau 1—before treatment, tau 2—after treatment, *CG*, control group)

	Sample 1 Mean ± SD tau (ng/ml)	Sample 2 Mean ± SD tau(ng/ml)	p sample 1 vs sample 2	Mean ± SD Sample 2/sample 1 ratio
Group 1	156.47 ± 119.698	304.44 ± 174.73	0.001	2.2 ± 1.18
Group 2	231.99 ± 177.618	527.81 ± 496.475	0.001	3.5 ± 5.43
CG	176.06 ± 81.215	n/a	n/a	
p Group 1 vs Group 2	ns	ns	n/a	ns
p Group 1 vs CG	ns	ns	n/a	n/a
p Group 2 vs CG	ns		n/a	n/a

We observed significant increase in tau concentration in sample 2 (after treatment) in both groups (*p* < 0.05).

The comparison between the group of patients who fully recovered and patients with sequelae (persistent symptoms observed 1 month after discharge) showed significant differences in tau concentration in both samples (Table 2) (*p* < 0.05).

**Table 2:** Comparison of tau concentration in two groups of patients: with full recovery and without full recovery

	Sample 1 Mean ± SD tau (ng/ml)	Sample 2 Mean ± SD tau (ng/ml)	p sample 1 vs sample 2	Mean ± SD sample 2/sample 1 ratio
No sequelae *n* = 21	129.62 ± 71.153	261.1 ± 152.68	0.001	2.11 ± 1.144
Sequelae *n* = 14	283.07 ± 188.42	604.65 ± 503.05	0.01	3.46 ± 5.459
p no sequelae vs sequelae	0.001	0.014		ns

**Fig. 1:**
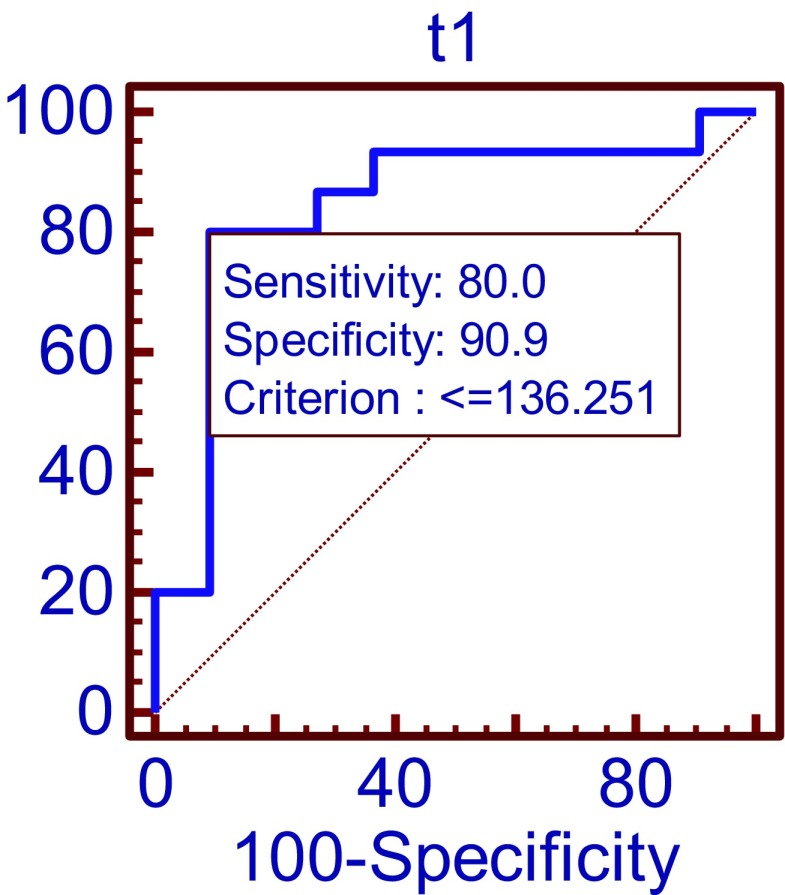
Comparison of tau protein concentration in sample 1 (before treatment) group of patients with sequelae and without sequelae by ROC curves *p =* 0.0001 AUC = 0.842

ROC curve analysis indicates that the cutoff at 136.25 ng/ml tau concentration in sample 1 may predict the sequelae presence with 90.9% specificity and 80% sensitivity (Fig. 1).

**Fig. 2:**
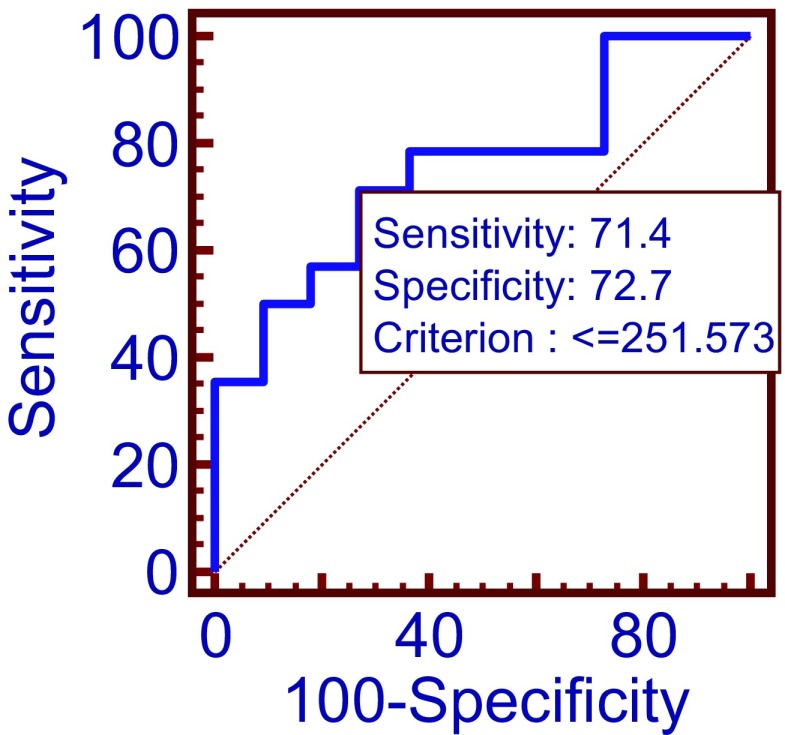
Comparison of tau protein concentration in sample 2 (after treatment) in group of patients with sequelae and without sequelae by ROC curves *p =* 0.01 AUC = 0.753

ROC curve analysis indicates that the cutoff at 251.57 ng/ml tau concentration in sample 2 may predict sequelae presence with 72.7% specificity and 71.4% sensitivity (Fig. 2).

Correlation analysis showed that in TBE patients (both groups) CSF pleocytosis (102 ± 66 cells/ml) in sample 1 correlated negatively with tau concentration in CSF (*R* = − 0.51, *p* < 0.05).

## Discussion

The frequency of sequelae development in TBE patients varies depending on the study from 20.6 to 63.6%. The most commonly reported symptoms were cognitive or neuropsychiatric complaints, balance disorders, headache, dysphasia, hearing defects, and limb paresis [9–11].

Patients with severe course of TBE (meningoencephalitis or meningoencephalomyelitis) are more likely to develop sequelae.

Factors predisposing to severe course or sequelae development in TBE patients have not been clearly established so far. Bogovic et al. reported that the severity of acute illness is positively associated with patient’s age, previous vaccination against TBE, peripheral blood leukocyte count, and serum CRP level, whereas it is negatively associated with the level of specific TBEV serum IgG antibodies [12]. The severity of the disease also depends on the host genotype (e.g., TLR3 gene or CD209 gene polymorphism) [12–14]. According to Czupryna et al., age and protein concentration in CSF were independent risk factors for sequelae development. Fowler et al. observed that high concentration of IFN-γ, IL-4, IL-6, and IL-8 in CSF might indicate a risk for incomplete TBE recovery in childhood. Additionally, the authors stated that the mechanism underlying the CNS pathology causing sequelae in TBE appears to be more likely related to the grade of CNS inflammation than to direct neuronal destruction [15].

Our previous studies proved that TBE infection in adults may lead to neurodegeneration. In the first study, we reported that patients with a history of TBE in MRI presented with cerebral atrophic lesions that could not be explained by age [16]. The other study proved that NSE concentration in CSF of patients with meningoencephalitis was significantly higher than in controls and in patients with meningitis; therefore, NSE concentration in CSF might be used for the prediction of sequelae development [3].

The results of our current study confirm that TBE may cause neuronal damage of CNS, expressed by increased tau concentration. Inflammatory process precedes the neurodegeneration as it was observed in our study where tau concentration was significantly higher in sample 2, even though the CSF inflammatory parameters (pleocytosis, protein concentration) in most cases remained normal. Tau concentration increased in both examined groups, yet it was significantly higher than in CG only in group II. The comparison of patients with sequelae and without sequelae showed that tau concentration in CSF was significantly higher in the sequelae group (in both samples 1 and 2). ROC curve analysis indicates that tau concentration in CSF might be considered as a biomarker of sequelae development in TBE.

The limitation of our study was a small number of patients included in the study, especially very small sequelae group.

## Conclusions

1. Neurodegeneration process is present in TBE encephalitis.
2. Tau protein concentration may be used as a predictor of complicated course of TBE.
